# Spatio-Temporal Dynamics of Hypoxia during Radiotherapy

**DOI:** 10.1371/journal.pone.0133357

**Published:** 2015-08-14

**Authors:** Harald Kempf, Marcus Bleicher, Michael Meyer-Hermann

**Affiliations:** 1 Department of Systems Immunology and Braunschweig Integrated Centre of Systems Biology, Helmholtz Centre for Infection Research, Braunschweig, Germany; 2 Frankfurt Institute for Advanced Studies, Frankfurt, Germany; 3 Institute for Biochemistry, Biotechnology and Bioinformatics, Technische Universität Braunschweig, Braunschweig, Germany; University of Dundee, UNITED KINGDOM

## Abstract

Tumour hypoxia plays a pivotal role in cancer therapy for most therapeutic approaches from radiotherapy to immunotherapy. The detailed and accurate knowledge of the oxygen distribution in a tumour is necessary in order to determine the right treatment strategy. Still, due to the limited spatial and temporal resolution of imaging methods as well as lacking fundamental understanding of internal oxygenation dynamics in tumours, the precise oxygen distribution map is rarely available for treatment planing. We employ an agent-based *in silico* tumour spheroid model in order to study the complex, localized and fast oxygen dynamics in tumour micro-regions which are induced by radiotherapy. A lattice-free, 3D, agent-based approach for cell representation is coupled with a high-resolution diffusion solver that includes a tissue density-dependent diffusion coefficient. This allows us to assess the space- and time-resolved reoxygenation response of a small subvolume of tumour tissue in response to radiotherapy. In response to irradiation the tumour nodule exhibits characteristic reoxygenation and re-depletion dynamics which we resolve with high spatio-temporal resolution. The reoxygenation follows specific timings, which should be respected in treatment in order to maximise the use of the oxygen enhancement effects. Oxygen dynamics within the tumour create windows of opportunity for the use of adjuvant chemotherapeutica and hypoxia-activated drugs. Overall, we show that by using modelling it is possible to follow the oxygenation dynamics beyond common resolution limits and predict beneficial strategies for therapy and *in vitro* verification. Models of cell cycle and oxygen dynamics in tumours should in the future be combined with imaging techniques, to allow for a systematic experimental study of possible improved schedules and to ultimately extend the reach of oxygenation monitoring available in clinical treatment.

## Introduction

Hypoxia plays a pivotal role in the development and progression of cancer, as it is directly connected to central phenomena such as quiescence, changes in the cell metabolism, altered motility and treatment resistance [1, 2]. As a consequence, the oxygenation of tumours is a major determinant for their aggressiveness, invasive potential and a predictive factor for the outcome of therapies [3–5]. While the persistent absence of oxygen in tumours can have negative effects on treatment, the natural and treatment-induced spatio-temporal dynamics of oxygenation also are of importance. Cyclic fluctuations of hypoxia have been shown to exert a strong influence on angiogenesis, radiosensitivity and gene expression of tumours [4, 6, 7].

This wide range of oxygen-related effects makes it desirable to include the oxygenation status of tumours in treatment planing. While hypoxia has a detrimental effect on radiotherapy and common chemotherapies, it can allow for the targeted use of hypoxia-specific therapies such as hypoxia-selective cytotoxins or bacterial lysis [8, 9]. Detailed information on the oxygen distribution within the tumour volume is thus necessary, in order to predict the effectiveness and schedule the exact combination of therapies.

An upcoming clinical tool for hypoxia detection is positron emission tomography (PET) which has a typical resolution of about 5mm in clinical settings [10]. As the hypoxic substructures in tumours are much smaller, this resolution limit implies that the micro-structure of hypoxia within a single voxel can not be effectively used to predict the therapy response and reoxygenation dynamics. Hypoxic areas in tumour tissue are often patterned on a scale of the inter-capillary distance of about 200–300 μm [11, 12]. Examples of this patterning have been observed with a range of different methods and in different tissues as for example in PC-3, EMT6 and 16C tumours [13], melanoma xenografts [14, 15] and in glioma (LN229) [16]. While the exact intercapillary distance can vary between tumour types and macroscopic regions, there is always an overall patterning as a results of the vascular structure. This self-similarity of macroscopic tumours makes it possible to study hypoxia by using tumour spheroids as a model for the tumour microregions in between capillaries. Spheroid cultures of cells posses a range of characteristics which correspond to *in vivo* tissues, most importantly the realistic mass transfer gradients of nutrients. Accordingly, they can be used as a model system to study basic reoxygenation dynamics in regards to the diffusion-based sources of hypoxia [17].

The imaging voxel size is of great importance as each voxel will contain a mixture of hypoxic and oxygenated cells, which will typically not be separated by resolutions in the order of 1mm. Tumour areas which appear to be non-hypoxic according to thresholding-methods can thus in reality contain significant hypoxic sub-regions which can drastically alter the treatment outcome [18]. As a consequence, the obtained PET data is partially unsuitable for the use in dose-painting calculations as it may lead to increased survival in hypoxic hot spots [18].

As neither patients nor tumours are static systems, insight into the oxygen dynamics in response to treatment is required on a microscopic and macroscopic scale, in order to identify local hypoxic areas and accordingly adapt the treatment schedules. Single measurements of the oxygen distribution or vascular structure are not sufficient for treatment planing due to the spatial and temporal dynamics of hypoxia [19]. Because of the current limits in temporal and spatial resolution, imaging of hypoxia can only assess the oxygenation on a coarse, thresholded level at few time-points during treatment, sometimes just characterising a whole tumour as “hypoxic” or “normoxic” [20]. This data basis is not satisfactory for efficient treatment design, because, as emphasised by this work, hypoxia is a phenomenon which has a pronounced structure on a scale below standard spatial and temporal imaging resolution.

Repeated hypoxia-imaging at selected time-points is required and must be combined with a model that can describe oxygen dynamics in the inter-imaging time periods and on spatial scales below the imaging resolution. Mathematical and computational modelling can provide the required insights and permit the investigation of the oxygenation dynamics under a wide range of assumptions. When combined with clinical imaging, the results of models can thus be used to include the oxygenation effects below the imaging resolution into treatment planing, and thus to complete the data basis for adaptive therapies until better imaging techniques become available.

### Sources, types and scales of hypoxia

The delivery of oxygen to tissue *in vivo* or *in vitro* has a multitude of facets. Three main mechanisms for the development of hypoxia can be coarsely distinguished: limited diffusion range, vessel occlusion and insufficient vascularisation [21].

**Fig 1:**
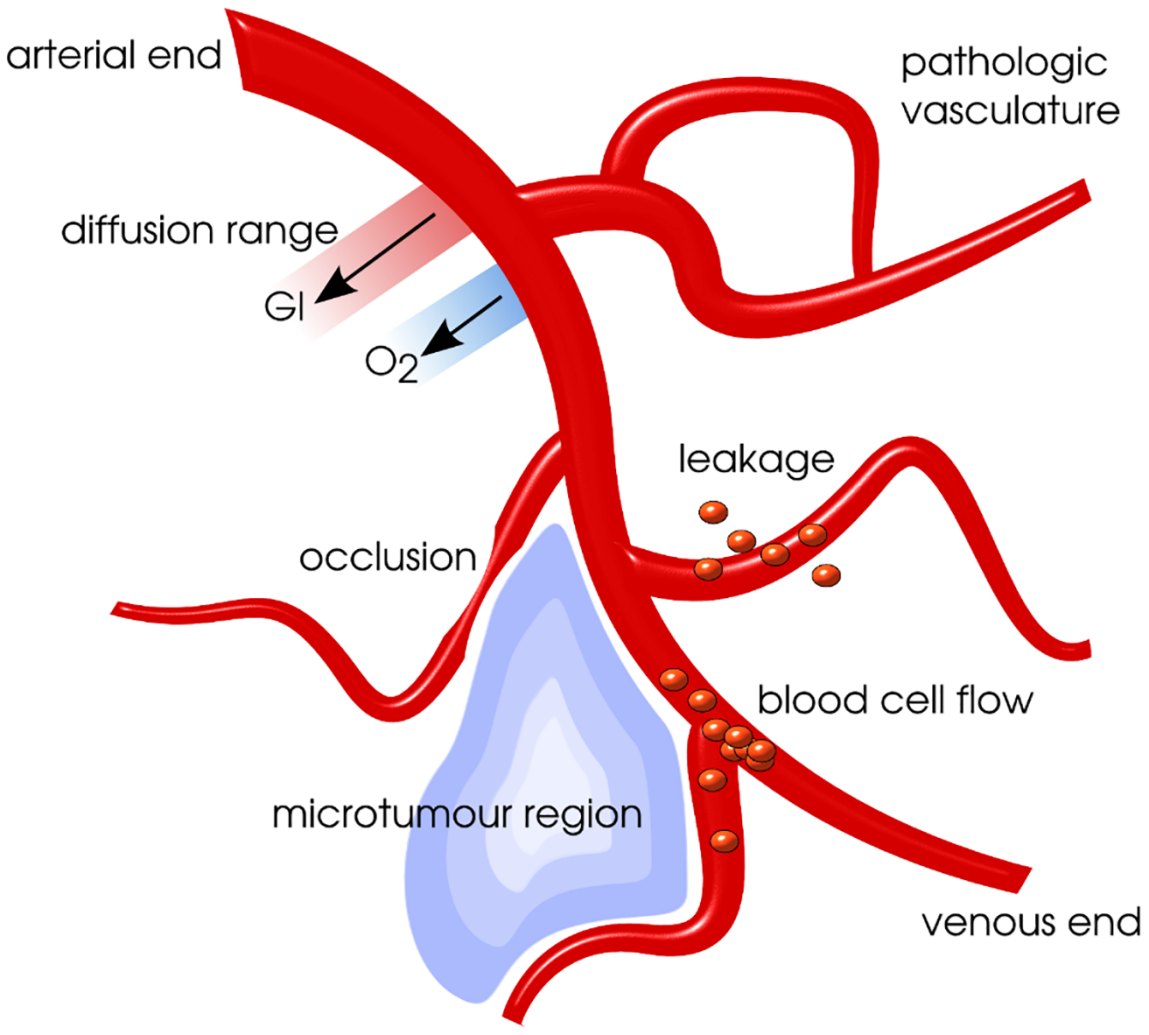
Hypoxia sources in tumours and diffusion range of substances. Illustration of selected hypoxia sources and a hypoxic tumour micro-region in tumour tissue. Hypoxic micro-regions will usually form a characteristic tiled pattern throughout a tumour. For *in vitro* examples of patterning in tumour xenografts see Fig 2 in reference [13].

The natural diffusion range of substances in a breathing tissue will lead to a critically low concentration of oxygen at a distance of more than approximately 100–200 from a capillary. If the distance is increased due to excessive growth of the tissue, which is not supported by the formation of new vessels, chronically hypoxic areas will form as illustrated in Fig 1. Within tumours the vascular density is known to be decreased with typical inter-capillary distances of about 300 μm [11, 12]. As these distances exceed the diffusion range, this leads to chronic hypoxia and a patterning of hypoxic regions with about 200 diameter [1, 13]. Suboptimal architecture of the vessels in angiogenesis can furthermore decrease the diffusion range and lead to a lacking supply in macroscopic regions of tissue [22].

Large parts of hypoxia dynamics are related to blood flow in the vascular network. As flow resistance in vessels is inversely proportional to the fourth power of the vessel diameter, small changes by vasomotion can cause large fluctuations in the network [4, 23]. Accordingly, vessel occlusions due to pressure, blood clotting, inflammation, and damage can furthermore influence blood flow and induce transient hypoxia also on a large spatial scale.

While the smallest spatial scale of hypoxia is on the order of 100 μm, temporal variations of pO_2_ in mouse models have been observed with periodicities of minutes, even in the absence of treatment [6]. Changes in perfusion, erythrocyte flux and vascular occlusion can occur within minutes and hours, while changes in the vascular network and angiogenesis usually take place on a scale of days. Fluctuations can be as extreme as maximal 18-fold changes in pO_2_ which have been observed with cyclic patterns on the scale of only minutes even in undisturbed tumours [7, 24].

Changes in vessel flow are hard to track and describe which makes acute, perfusion-driven hypoxia unpredictable [25]. The diffusion-based hypoxia on the other hand can be investigated in spheroid systems and modelled in a straightforward approach.

### Effects of hypoxia on therapy and treatment planning

Oxygen is of special importance for the effectiveness of radiotherapy, as radiation damage to cells is drastically reduced under hypoxic conditions [25]. This oxygen enhancement effect (OER) is present in every tumour region of low oxygen concentration and will only vanish above an oxygen partial pressure of about 10 mmHg pO_2_, a concentration which is typically used as definition of the hypoxia threshold [26]. The according oxygen modification of radiosensitivity for the clinical standard, the linear-quadratic model (LQ), was first proposed in 1956 [27] and is now commonly used in treatment planing [25, 28]. While oxygen enhancement is especially important for radiation with low linear energy transfer (LET) such as x-rays (with a typical LET of 2keV/μm), it is also relevant in modern therapies which employ high-LET radiation in extended Bragg peaks [29].

In order to cancel out the effects of oxygen-shortage, the dose to hypoxic tumour regions is often increased in radiotherapy plans or modified in dose-painting approaches. This approach however depends critically on the spatio-temporal resolution of information on the oxygen kinetics. As the location of hypoxic areas is likely to change on a day-to-day basis, prescription plans might already be invalidated as soon as they are started, if the resolution is not sufficient [30].

As the action of a wide range of drugs will also depend on the availability of oxygen, the importance of oxygen dynamics in treatment planing extends also to radio-chemo schedules and pure chemotherapy [3]. Furthermore, in drug delivery the treatment-induced changes in diffusivity of the tumour tissue will be of great importance [31].

### Detection and measurement of hypoxia

As the success of therapies to a large degree depends on an accurate imaging of hypoxia, the detection and quantification of hypoxia is continuously becoming more precise and cost-efficient [32, 33]. For *in vitro* experiments a range of methods such as polarographic needle measurements or immunostaining allow for dynamic, quantitative or large-scale assessment of hypoxia in tissue. While some of these techniques are also suitable for *in vivo* use, the availability of techniques is more limited, especially if non-invasive methods are preferred [34, 35].

Positron emission tomography (PET), as a clinical tool for hypoxia detection, suffers from both relatively low spatial and temporal resolution. Low tumour to blood ratios of tracers and long scan times limit the temporal resolution to 3 hours [30]. This implies that the time-point of PET observation is critical for the assessment of the true oxygenation, especially in tumours under the effects of treatment [10]. Relatively long PET scan times lead to motion averaging of heterogeneity and fast tumour hypoxia. Also spatially the resolution of PET imaging is quite limited. A clinical resolution of about 5 mm^3^ voxel size is typical, while advanced systems will be able to achieve resolutions of 1 mm^3^ [10].

In summary, PET measurements can usually only provide integral, time-averaged values of local oxygen concentration and will suffer from blurring effects and background noise during imaging. As a consequence, they cannot capture the micro-structure of hypoxia, which is below the typical clinical resolution, but which is of vital important for the response to therapy and the subsequent reoxygenation dynamics.

Newer imaging techniques, as for example electron paramagnetic resonance imaging (EPRI), can provide absolute oxygen concentrations from three-dimensional systems with a resolution of 1 mm and 2 min [6]. However, EPR imaging is currently not employed in clinical settings and will require further development to become widely available. In an experimental setting, EPR imaging allows for extreme temporal and spatial resolution which can be used to assess the predictions of oxygen dynamics models and to increase their accuracy by tuning of model parameters. Furthermore, a range of innovative high quality imaging and measuring techniques are under development [36, 37], which facilitate data gathering in spheroids and make it possible to image in *in vivo* systems [38, 39].

For clinical practice, a non-invasive, high-frequency, quantitative imaging technique is desirable, in order to track hypoxic dynamics over minutes and days [6]. Lin and Hahn call for a repeated combined PET/CT imaging during therapy in order to verify the tumour response in hypoxia and to use the information for an accurate dose painting and escalation in acutely hypoxic regions [30].

A connection between imaging and modelling has to be established in order to predict oxygen dynamics beyond available imaging resolution. An example of an observable which can connect model predictions of hypoxia dynamics and experimental observations is the uptake of tracers in PET-imaging [30].

### Predicting oxygen dynamics *in silico*

The chronic hypoxia in a tumour spheroid is spatially similar to hypoxia in an inter-capillary microregion of tumour tissue, as is illustrated in Fig 1. Accordingly, within the current work, the modelling of hypoxia is not performed on the level of averaged voxels and with timesteps of hours, but instead for a small nodule with a diameter of 1000 μm. Thus the system provides an intrinsic dynamic of tumour hypoxia, which is in the same form repeated as a pattern within the inter-vascular spaces of *in vivo* tumours.

This approach is drastically different from alternative *in silico* models of tumour hypoxia which typically employ averaged 1D cell populations [40, 41], 2D lattice-based cellular automata [42, 43], restrain themselves to analytical solutions of oxygen availability [44] or known micro-vessel distribution [45]. A range of models has addressed the issue of tissue oxygenation measurement with polarographic needles in regards to reliable and accurate measurements [46, 47], while relying on a known, pre-imposed or measured vascular network. Also the effects of the vascular network itself have been studied in regards to growth-inhibiting and -promoting effects [48].

Most existing models for tumour hypoxia make use of large, averaged voxels in order to represent a subset of individual cells, are restricted to a regular grid with rather low cell numbers (often in the order of only 10000 cells), impose strong symmetry assumptions to derive analytical solutions or use extremely large timesteps on the order of hours or days [49]. In contrast to this approaches our model will employ a lattice-free 3D environment, in which a high number of individual cells (up to 1000000) is represented on a timescale of seconds and can interact with a high-resolution diffusion solver of 71^3^ grid nodes, which also takes into account the local variability of the diffusion coefficient in dependence on the cell density. A range of alternative high-performance on- and off-lattice models could be suitable targets to be employed in a similar type of hypoxia modelling by introduction of nutrient diffusion into the according models [50, 51].

We will address the previously unregarded timescale of tumour hypoxia and oxygenation in the range of minutes and hours and show that treatment-induced changes will affect the outcome in fractionated radio- or chemotherapy treatment protocols. Our focus will rest on the local, high-resolution dynamics of oxygenation both in space and time. In contrast to the usual perception, we will demonstrate that the “chronic” diffusion-limited hypoxia can lead to fast and drastic changes in tumour oxygenation, which are—to a large degree -predictable in contrast to other sources of hypoxia.

## Methods

### Cell-based spheroid growth model

A three-dimensional, single-cell based model is used in order to track the growth of tumour spheroids from a small number of seeder cells and to monitor their oxygenation response in reaction to therapeutic approaches [52, 53]. The spatial arrangement of cells as tissue is represented in a Voronoi-Delaunay model [54–56] and the adhesive-repulsive interaction between cells is modelled using the Johnson-Kendal-Roberts approach [57, 58]. The simulation relies on experimentally measured cell parameters from EMT6 and V79 cells in order to achieve a realistic implementation of a generic cancer cell.

**Fig 2:**
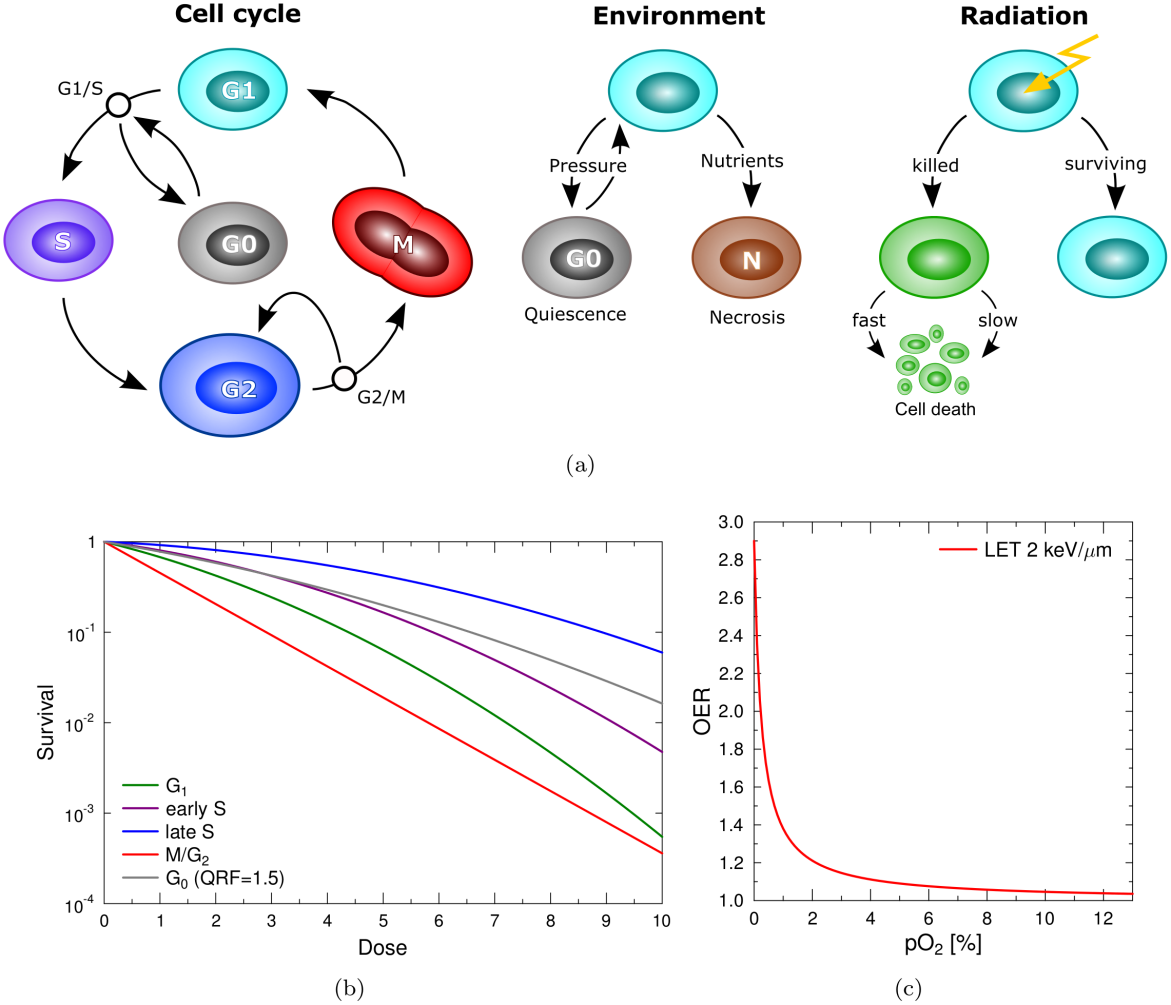
Regulation of the cell cycle and radiation reaction. (**a**) Overview of the cell cycle regulation, cell responses to environmental factors and radiation. (**b**) Survival curves for cells in specific cycle phases and with increased radioresistance due to quiescence, as described with the linear-quadratic model Eq 5. (**c**) Dependency of the oxygen enhancement ratio on the local oxygen concentration employed in order to alter the radiation response for hypoxic cells according to Eq 6, calculated according to equation 8 from [59].

Cells in the simulation are subject to a cell cycle regulation, as shown in Fig 2(a), and will accordingly react to the realistic nutrient and pressure gradients that develop in the growing tumour spheroid. Quiescence of cells is by default triggered if a cell is subject to a critically high pressure *P*_crit_ of more than 200 Pa from neighbouring cells at the G1/S-checkpoint. The cell is released back into the active cycle once the pressure decreases to sub-critical levels. Cells are considered to be fully tolerant to anoxia during the total time of modelling. In contrast to this, a local depletion of glucose leads to the starvation of cells and accordingly triggers necrosis.

### Cell nutrient consumption

Cells consume oxygen and glucose from a diffusion system which is superimposed on the spatial tumour model. As the nutrient uptake is more regulated by metabolic needs than by environmental factors, nutrients are taken up by a cell independently from the concentration. This implies zero-order uptake kinetics, in which cells consume a constant amount of nutrients per time interval, according to experimentally measured consumption rates (as indicated in Table 1 [52, 60]).

**Table 1:** Listing of parameters used in the model. Further parameters and sources for cell interaction, nutrient diffusion and consumption can be found in [52] and [56].

**Parameter**	**Value**	**Sources and remarks**
Diffusion nodes	71	per dimension
System size	1000 μm	cubic lattice
Glucose boundary conc.	5 mM	capillary blood
Oxygen boundary conc.	0.13 mM	[64]
Update intervall	10 min	
Glucose diffusion coefficient water $D_{{\text{H}}_{2}0}^{\text{G1}}$	690 μm^2^/s	[65]
Glucose diffusion coefficient tissue $D_{\text{Tis}}^{\text{G1}}$	105 μm^2^/s	[66]
Oxygen diffusion coefficient water $D_{{\text{H}}_{2}0}^{{\text{O}}_{2}}$	3300 μm^2^/s	[67]
Oxygen diffusion coefficient tissue $D_{\text{Tis}}^{{\text{O}}_{2}}$	1750 μm^2^/s	[67]
Initial cell radius (start G1)	7.94 μm	[63]
Final cell radius (end G2)	10 μm	[63]
avg. necrosis and apoptosis duration τ*_n_*, τ*_A_*	24 h, 12 h	[68–70]
Starvation glucose level	0 mM	growth fit
Quiescence critical pressure *P*_crit_	200 Pa	[71], growth fit
Hypoxia threshold	0.014 mM	[26]
Active glucose consumption	180amol cell^−1^ s^−1^	[60, 63]
Quiescent glucose consumption	130amol cell^−1^ s^−1^	[60, 63]
Active oxygen consumption	83amol cell^−1^ s^−1^	[63]
Quiescent oxygen consumption	49amol cell^−1^ s^−1^	[63]
acute death chance AC	0.66	[52] [53]
mitotic mismatch rate MM	0.3	[52] [53]
LQ-model *α*_G1_, *β*_G1_	0.351, 0.04	[25, 72]
LQ-model *α*_S_, *β*_S_	0.1235, 0.0285	[25, 72]
LQ-model *α*_M_/_G2_, *β*_M/G2_	0.793, 0	[25, 72]
Quiescence resistance factor QRF	1.5	[73, 74]
Maximum oxygen enhancement *O*_max_	2.9	[29]
OER half-life concentration	0.0052 mM	[25]

For cells in the quiescent state the oxygen consumption has been demonstrated to be decreased [61, 62]. To capture the reduced metabolic needs of quiescent cells, their consumption rates for oxygen and glucose in the model were decreased accordingly as measured in [63] and indicated in Table 1.

### Diffusion modelling

Nutrients enter the spheroid by diffusion through its boundary surface and are locally consumed by cells, which can be described by the diffusion equation with sink term *Q*(*x*, *t*)

$$\begin{array}{ccc} \frac{\partial C(x,t)}{\partial t} & = & \frac{\partial^{2}}{\partial x^{2}}(D(x,t)C(x,t))+Q\left(x,t\right). \end{array}$$ (1)

The boundary surface is kept at a constant nutrient concentration, as a sufficiently well-mixed reservoir of nutrients is assumed to be available for support from the blood stream. As the tumour changes in size and shape, the boundary surface is adapted so that any node outside the tumour which is not occupied by cells will become part of the boundary. While a common simplification in diffusion modelling is the assumption of a constant diffusion coefficient [47, 75], it is necessary to consider a local variation of the diffusion coefficient. As the diffusion coefficient for substances in water differs greatly from the diffusion in tissue, this step is vital in order to obtain a reliable result. This is especially true when the integrity and density of the tissue under observation will be altered by therapeutic approaches, due to dissolution of dead cells. As a consequence, the diffusion coefficient within the system is space-dependent and will vary between the measured coefficients for diffusion in water *D*_H_2_O_ and tissue *D*_Tis_

$$\begin{array}{c} D\left(x,t\right)=D_{\text{Tis}}+\left(D_{{\text{H}}_{2}\text{O}}-D_{\text{Tis}}\right)\frac{\varrho \left(x,t\right)}{d_{\text{max}}} \end{array}$$ (2)

depending on the local cell density *ϱ*(*x*, *t*) (calculated as cells per volume) and the maximum density in a dense packing *d*_max_ (determined from a compressed cell mass in the system). This approach is a novelty and improvement of the diffusion system in comparison to other diffusion models. A linear dependency of the diffusion coefficients on local density is assumed as first approximation. With this addition the diffusion equation of the system becomes

$$\begin{array}{ccc} \frac{\partial C(x,t)}{\partial t} & = & \frac{\partial }{\partial x}(D\left(x,t\right)\frac{\partial C(x,t)}{\partial x})+Q\left(x,t\right)\; \end{array}$$ (3)

where for the steady state case the assumption $\frac{\partial C(x,t)}{\partial t}\approx 0$ is valid. This leaves a Poisson-type problem for the system

$$\begin{array}{ccc} \frac{\partial }{\partial x}(D\left(x\right)\frac{\partial C(x)}{\partial x}) & = & -Q(x). \end{array}$$ (4)

This reaction diffusion equation is numerically solved in a finite difference form, using the Crank Nicholson scheme under careful implementation of mechanisms, which guarantee the validity and stability of the solution, such as an adaptive maximum stepsize and sanity checks. By default, a pseudo-steady-state approximation is employed in order to recalculate the steady-state of diffusion in fixed time steps using an unconditionally stable biconjugate gradient method. This approximation is possible as the non-stationary evolution time of the system is very short [45, 76].

The diffusion of glucose and oxygen is modelled on a cubic reaction diffusion solver system of 1 mm edge length using 71 nodes per dimension. Any off-grid concentration is retrieved or stored in the solver using trilinear interpolation. Nutrient conditions in regions of the system which are not occupied by cells are fixed to typical concentrations in arterial blood of 5 mM glucose and 0.13 mM oxygen [64]. For diffusion of substances in water and tumour tissue at 37°C the following coefficients are used: $D_{{\text{H}}_{2}\text{0}}^{\text{G1}}=690\;{\mu \text{m}}^{2}/\text{s}$ [65], $D_{\text{Tis}}^{\text{G1}}=105\;{\mu \text{m}}^{2}/\text{s}$ [66] (EMT6 spheroid), $D_{{\text{H}}_{2}\text{0}}^{{\text{O}}_{2}}=3300\;{\mu \text{m}}^{2}/\text{s}$ [67], and $D_{\text{Tis}}^{{\text{O}}_{2}}=1750\;{\mu \text{m}}^{2}/\text{s}$ [67] (DS-Carcinosarcoma *in vivo*).

### Reactions to irradiation

The total amount of cell death in response to a radiation dose is matched to experimental observations via the linear quadratic model for single cell survival [25, 28]. Cells use a representative *cell cycle phase-dependent* survival probability *S*_*p*_ to assess their survival in dependence of the radiation dose *D*:

$$\begin{array}{c} S_{p}=e^{-(\alpha_{p}D+\beta_{p}D^{2})} \end{array}$$ (5)

As physiological example, *α*_*p*_ and *β*_*p*_ values of V79 hamster cells subjected to x-ray irradiation by Sinclair [72] are employed, as shown in Fig 2(b) (see also Table 1). These values reflect the typical behaviour of cells exemplarily, with high radioresistance in S-phase and high radiosensitivity in G2/M-phase. Since the radiation sensitivity of quiescent cells has been demonstrated to be lower than that of actively cycling cells, the effective dose to cells which are in quiescence is scaled down in the model using the quiescence resistance factor QRF = 1.5 [73, 74].

Cells committed to radiation-induced death are either killed immediately via an acute, fast death process (fatal damage with probability *AC*) or later in their cycle progression by delayed death (e.g. in response to mitotic catastrophe), as indicated in Fig 2. While marked as damaged, a cell is arrested once it reaches the G2/M checkpoint until it progresses to cell death with a “mitotic mismatch”-rate *MM*.

The radiosensitivity of cells decreases for critically low oxygen concentrations below 20–30 mmHg [25, 77]. These effects of local oxygenation on radiation damage are taken into account via the oxygen enhancement ratio (OER), which is formally defined as the ratio of doses needed to achieve a certain survival level in cells, either in normoxic conditions *D*_ox_ or under hypoxic conditions *D*_hy_:

$$\begin{array}{c} \text{OER}\left(pO_{2}\right)=\frac{D_{\text{hy}}}{D_{\text{ox}}}{|}_{\text{same}\;\text{effect}}\;=\frac{O_{\text{max}}\;\cdot \;pO_{2}+H}{pO_{2}+H} \end{array}$$ (6)

Eq 6 implies a dependency which is determined by the maximum OER effect *O*_max_ and the pO_2_ at half effect *H* in the commonly used parametrisation provided by Alper and Howard-Flanders [27]. The according maximum OER in V79 spheroid cells was measured to be 2.9 for X-rays (250kV) with an LET of 1.7keV/μm [29]. The employed oxygen enhancement for the simulation is shown in Fig 2(c) and calculated according to equation 8 from [59]. The half-life of oxygen enhancement is around 0.5% pO_2_ [25]. The OER varies only mildly throughout the cell cycle between 2.3 for G2-cells and 2.8 for S-cells and consequently has been assumed to be cycle-independent.

### Measuring of hypoxia and reoxygenation

The oxygenation status of all cells in the simulation is available as observable at all times. One of the measurables under investigation is the fraction of cells which are subjected to a local oxygen concentration below a defined threshold. Spatial profiles of oxygen concentration and the temporal evolution of the concentration at the centre of the tumour spheroid are also of interest, as they contain information on the localisation of hypoxic cells.

One of the non-invasive techniques employed in clinical settings is the monitoring of the oxygen concentration in the tumour via PET, which will typically yield spatially resolved images of the tumour volume with a voxel size of about 5*mm* ^3^. Below this level only time and space-averaged data is usually obtained [10]. Consequently an integral measure of oxygen

$$\begin{array}{c} P\left(t\right)=\int_{\mathrm{Tumour}}C\left(x,t\right)\;d^{3}x\; \end{array}$$ (7)

can be used to compare model predictions with experimental and clinical results.

The observable *P*(*t*) is directly related to the oxygen-dependent tracer-quantity which is measured in PET-monitoring of tumour-subregions.

## Results

### Spheroid growth and rise of hypoxia

**Fig 3:**
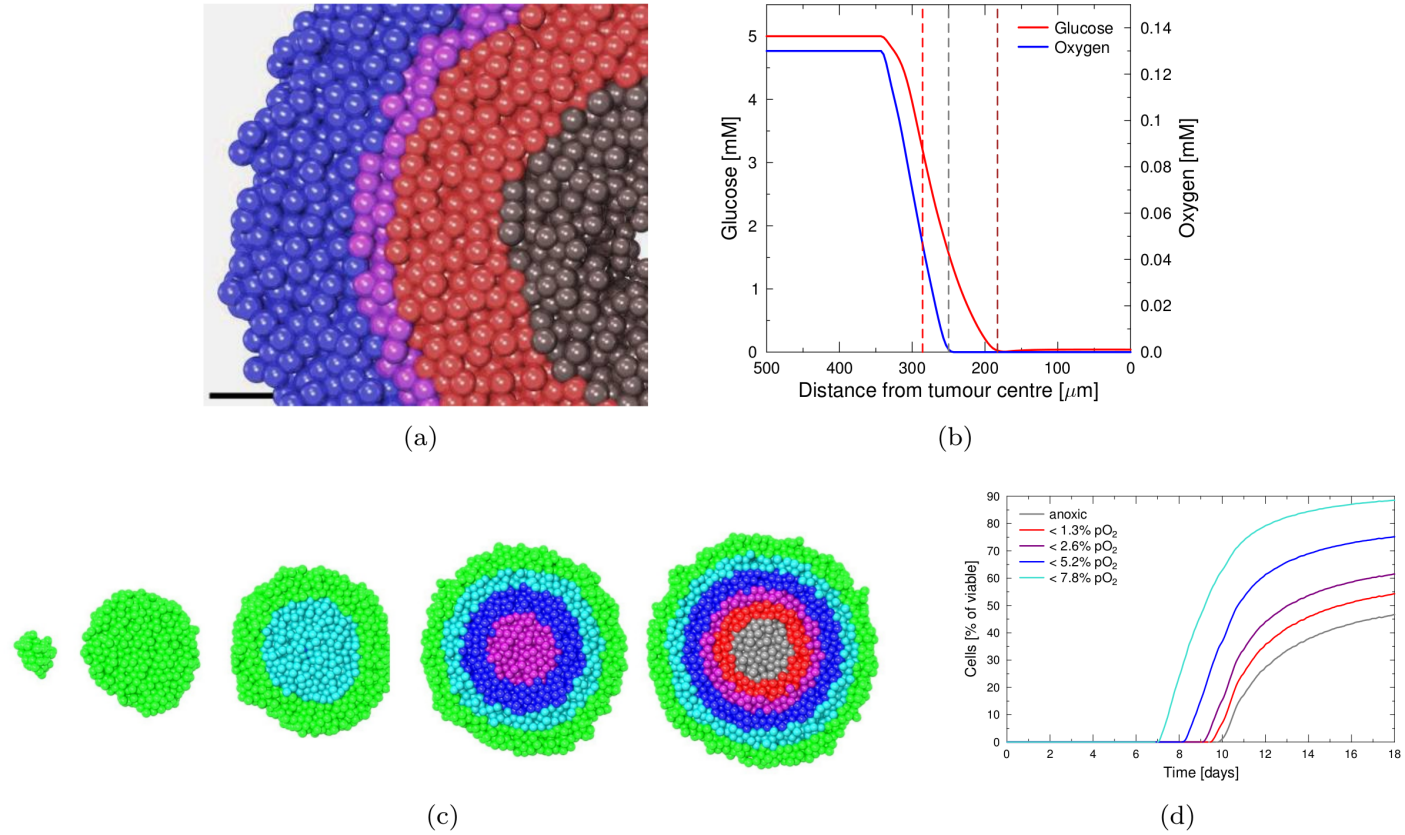
Distribution of nutrients in the spheroid and de-oxygenation during growth. (**a**) Central cut-section through an *in silico* EMT6 tumour spheroid after 12 days of growth. Normoxic cells (pO_2_ > 2.5%) in blue, cells below 2.5% pO_2_ in purple, below 0.5% in red and necrotic cells in grey. Slice thickness is 40 μm. Compare to the *in vitro* immunodetection of pimonidazole in hypoxic cells in the peri-necrotic regions of a tumour spheroids (T47D after 10–14 days of growth) in Fig 3a in [80] (bar size in both images 50 μm). (**b**) Lateral concentration profiles of glucose and oxygen. The higher reach of survival-promoting glucose will lead to the formation of an anoxic region (between grey and brown dashed line). Hypoxic cells will be found in low-oxygen regions (between red and grey dashed line) and normoxic cells beyond the red dashed line. Compare also reference [1]. (**c**) Central cut-section of 40 μm thickness through an *in silico* EMT6 tumour with oxygen-dependent staining after 2, 6.5, 8.3, 9.5 and 10.2 days of growth. Cell staining corresponds to the line color in graph (d) (green cells are above 7.8%pO_2_). (**d**) Development of cell populations below defined oxygen concentrations in the spheroid as visualised in (c).

*In silico* spheroids were grown from 10 seeder cells, which were initially distributed randomly in the gap and synthesis phases of the cell cycle. Spheroids grew to a typical diameter of 700 μm after 14 days of proliferation. Steep oxygen and glucose gradients developed inside the spheroids as visualised in Fig 3. After about 9.5 days of growth, a hypoxic population developed as a result of the limited diffusion range of oxygen, followed by central anoxia after 10 days. Total glucose deprivation in the centre of the spheroid was observed after about 11 days and led to the onset of central necrosis (see Fig 3(c) and 3(d)). 12 days after seeding the relative distribution of cells in the cell cycle phases, as well as the oxygenation was sufficiently stable in order to study the reaction of the spheroid system to irradiation.

The observed diffusion range of substances in the spheroid corresponded well to experimental measurements and theoretical predictions (100–200 μm range *in vivo* [78, 79], 200 μm range of O_2_ predicted [75]). Fig 3(b) shows the diffusion range of oxygen and glucose in the spheroid at the 14th day of growth. While the central region was void of both oxygen and glucose, a large layer of viable but anoxic cells developed due to the larger range of survival-promoting glucose. A steep concentration gradient led from a hypoxic cell population up to the normoxic population at typical capillary blood concentrations on the surface of the spheroid.

A qualitative comparison of hypoxia *in silico* and *in vitro*, as in Fig 3(a) and Fig. 3a in [80], illustrates the histological correspondence of the tumour spheroid model to experimental spheroid systems.

### Response to treatment and reoxygenation dynamics

**Fig 4:**
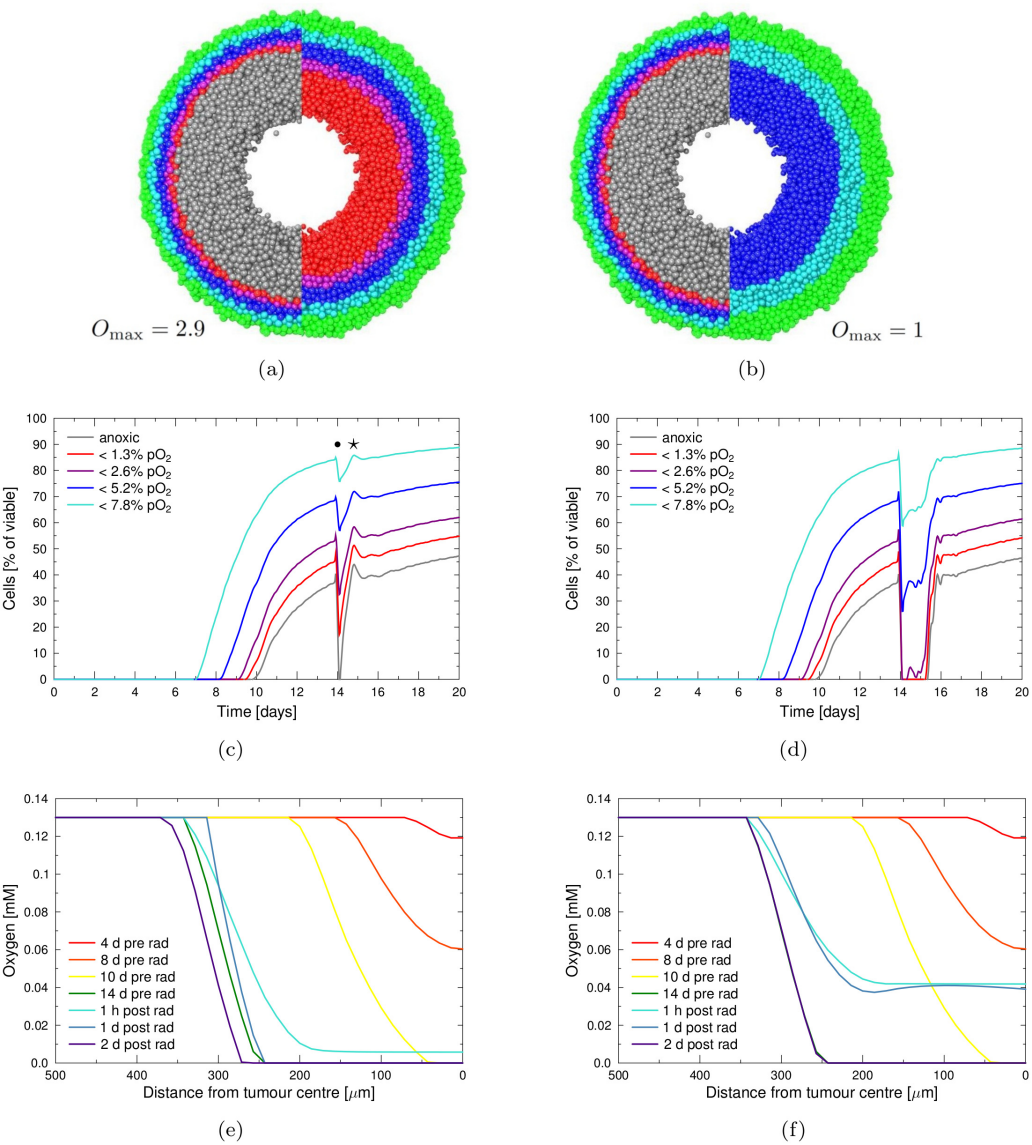
Reoxygenation of the spheroid in response to a single radiation dose. Irradiation has been performed with a dose of 4 Gy at day 14, either with normal oxygen enhancement (*O*_max_ = 2.9, left column) or with suppressed oxygen enhancement (*O*_max_ = 1, right column). (**a-b**) Pre- (left) and post-radiation (right) cutsection with oxygenation staining of cells as in Fig 3(c) and 3(d). (**c-d**) Fraction of cells which are subjected to different oxygen levels during growth and irradiation. Dot marker shows treatment-induced increase in hypoxic cells and star marker the regrowth-induced increase (refer to main text for details). (**e-f**) Lateral concentration profile of oxygen in the tumour spheroid at different time points during growth and treatment.

**Fig 5:**
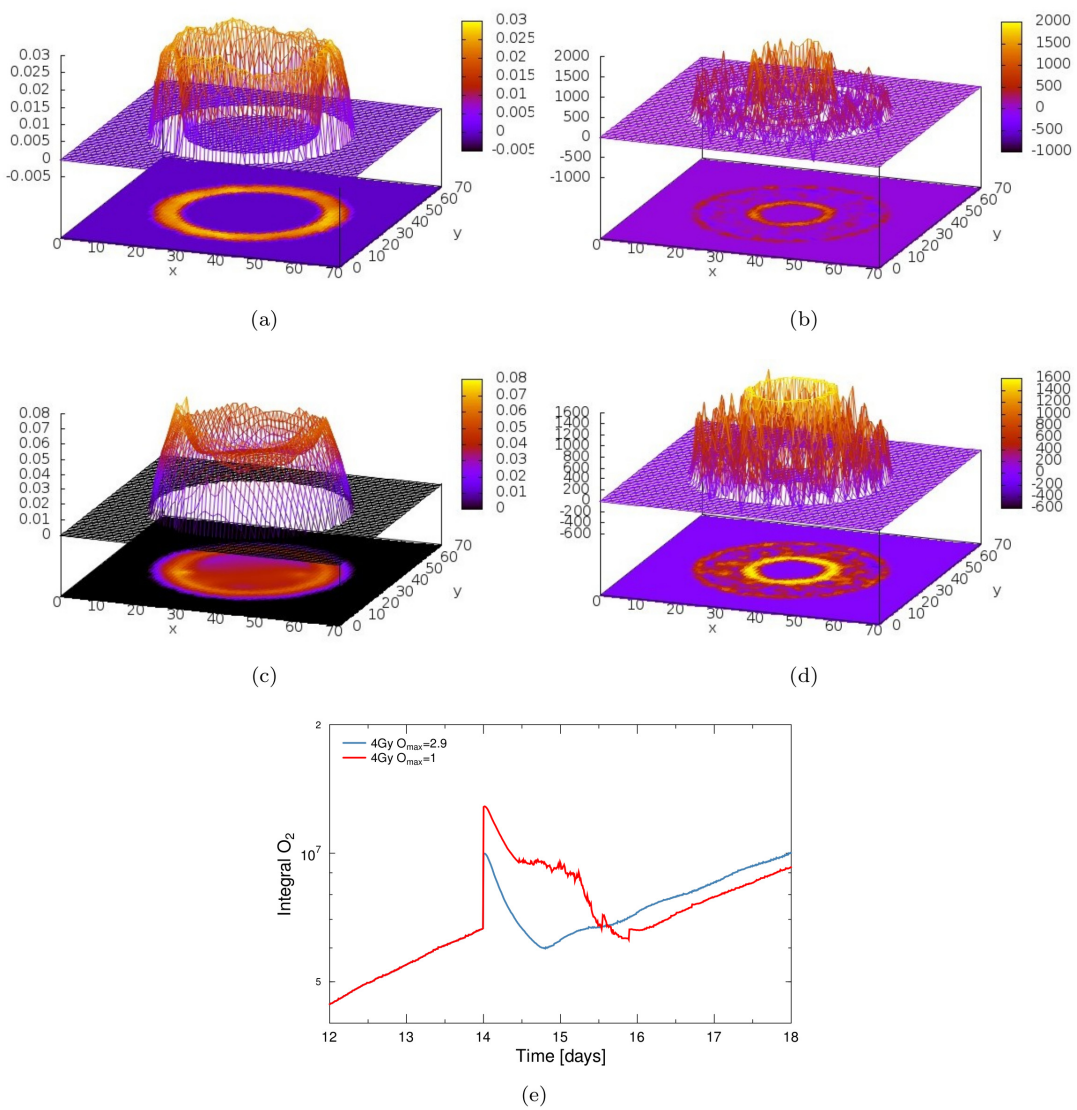
Changes in oxygenation and diffusivity in response to irradiation and integral oxygen concentration as PET-equivalent. (**a-d**) Change in the oxygen concentration (left column, difference in mM) and the density-dependent oxygen diffusion coefficient (right column, difference in μm^2^/s) between a pre- and post-radiated tumour state. Visualisation shows the difference in a central cutslice of the diffusion system (x and y denote grid nodes over 1000 μm, z and the color palette illustrate the difference in the according units). (a-b) show the rise in local oxygen concentration and the increase in the diffusivity as difference between a pre-radiated state and 12 hours after irradiation with 4 Gy and *O*_max_ = 2.9, (c-d) for 4 Gy and *O*_max_ = 1 with 24 hours difference. The dissolution and permeabilisation of dead cells will lead to a significant rise of oxygen diffusion into the tumour bulk. This influx is in parts due to a lower local consumption but also due to local increase in tissue diffusivity. (**e**) Integral oxygen *P*(*t*) in the tumour spheroid in response to a single irradiation dose of 4 Gy x-rays at day 14 with full or suppressed oxygen enhancement in effect. The integral signal can be quantitatively compared to PET signals.

Irradiation with 4Gy of x-rays drastically affected the oxygenation of the tumour and triggered a complex cascade of reactions, as illustrated in Fig 4. Cell death in response to the radiation dose led to a significant drop in the nutrient consumption density, which allowed for a far greater penetration depth of nutrients. In addition, the dissolution of dead cells decreased the tissue density and consequently led to a higher diffusion coefficient, which further increased the range of oxygen and glucose, as shown in Fig 5.

The observed increase in penetration depth of diffusing substances is illustrated in Fig 4(a). While directly prior to irradiation a large anoxic and hypoxic population was present in the inner region of the spheroid, anoxia was completely lifted already one hour after irradiation. The width of the cell layers which were subjected to high concentrations of oxygen accordingly increased throughout the tumour volume.

The dissolution of dead cells also led to a drop in the pressure on quiescent cells, which allowed for the re-entry of previously non-cycling cells into the active cycle. Through the associated increase in nutrient consumption, a fast re-depletion of nutrients was triggered.

As a result, the typical hypoxic fraction of cells drastically changed in response to irradiation. A slight increase in the fraction of hypoxic cells immediately after irradiation was observed, which was due to radioresistance of hypoxic cells that translated directly into a survival advantage (see dot marker in Fig 4(c)). As expected, this temporary increase was relatively higher for populations with lower oxygen concentration. In general, a full re-oxygenation of the tumour was observed. However, the concentration in inner regions of the spheroid was not lifted above the hypoxic threshold by a radiation dose of 4Gy, as is shown in Fig 4(e). Within 4 hours an anoxic population of 5% had reformed and after 15 hours levels of oxygenation were reached which were comparable to the pre-treatment situation. Due to the strong regrowth of the spheroid, an overshooting of consumption was observed, which decreased the availability of oxygen even below pre-treatment levels 19 hours post-radiation (see star in Fig 4(c)).

If the maximum oxygen enhancement ratio was set to unity (*O*_max_ = 1), thus mimicking the use of hypoxic sensitizers and effectively making radiation damage oxygen-independent, the reoxygenation response was drastically enhanced (compare Fig 4(a) and 4(b)). As hypoxic cells in the central regions of the spheroid were no longer subject to a survival advantage from their increased radioresistance, a higher fraction of cells was killed by irradiation.

The following increase in diffusivity and decrease in consumption led to a significantly higher influx of oxygen and a longer persistence of reoxygenation in the tumour. Consequently, irradiation with 4Gy fully re-oxygenated the tumour to a level which was well above the hypoxia threshold of 0.014 mM throughout the whole spheroid, as is shown in Fig 4(f). The reoxygenation persisted for 42h before pre-treatment levels of anoxia were reached again. Regrowth would at the same time increase the consumption density and decrease the diffusion coefficient, so that the re-depletion of oxygen was a very fast process.

Especially in the case of *O*_max_ = 1, local fluctuations in oxygenation could be observed, which were driven by the interplay of the re-entry of quiescent cells into the active cycle, the associated tumour regrowth, local nutrient depletion and necrosis. This dynamics led to a complex development of local oxygenation, as is visible in Fig 4(d) and visualised in S1 Fig.

### PET comparison and optimal imaging

If local invasive techniques are available for assessment of the oxygenation during treatment, then the oxygenation profile can be compared quantitatively as in Fig 4(e). For non-invasive techniques on the other hand, only integral oxygen concentrations can be used for comparison.

Fig 5(e) shows a sample of the integral oxygen dynamics within the tumour spheroid. Radiation triggered a fast re-oxygenation in the tumour, which was expressed as a sudden rise in the integral amount of oxygen. If the typical oxygen enhancement effects were present, a smooth re-depletion of oxygen was observed over 16 hours. With suppressed oxygen enhancement the re-depletion signal was more complex and extended over almost 48 hours, due to severe damage to cells also in hypoxic areas.

Due to the micro-structure of the tumour tissue, the macroscopic, diffusion-related change in the PET-signal will contain a component which is a direct result of the change within each micro sub-volume (which in turn corresponds to the modelled tumour spheroid *in vitro*). Thus a part of the observed change in the PET-signal can be predicted and compared directly to experimental measurements.

While the local oxygen concentration naturally showed extreme fluctuations in response to irradiation, the integral oxygen measure also exhibited dynamics on a short timescale, as is visualised in Fig 5(e). This illustrates the critical importance of imaging timing, as assessment of oxygenation at transient extreme points can lead to false dose escalation and treatment planning.

### Persistence of reoxygenation during repeated treatment and its impact on sequential therapies

Reoxygenation during the course of fractionated therapies is of great interest for treatment planing. Ideally, therapies achieve a lasting reoxygenation within a short time, as this enables following fractions to exploit the enhancing effects of oxygen. To allow for this effect, the reoxygenation must be persistent for a time period which is equal to, or greater, than typical clinical scheduling intervals.

Within the simulations the oxygenation response was identical upon repeated irradiation, provided that the interval between fractions was chosen larger than the typical time needed for re-establishment of a pre-treatment population of hypoxic cells. If treatment intervals were chosen smaller, consecutive fractions of radiation each affected a tumour which was not fully in equilibrium, but slightly more oxygenated than at the start of treatment. In consequence, a build-up of a lasting oxygenation could be achieved.

**Fig 6:**
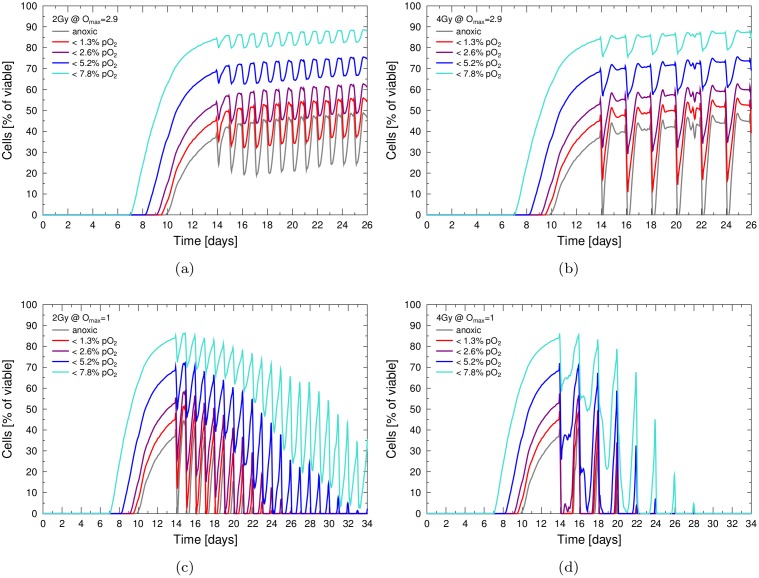
Reoxygenation during repeated radiation delivery. Persistence and stability of the reoxygenation response during multiple fractions and in prolonged irradiation regimes. Tumours where either irradiated with doses of 2 Gy per 24h (left column) or 4 Gy per 48h (right column). While a stable reoxygenation response can be observed, full reoxygenation is only possible if oxygen enhancement effects are suppressed via *O*_max_ = 1 as shown in the bottom row.

Typical scenarios for repeated radiation delivery are fractions of 2Gy every 24h in a conventional radiation schedule or 4Gy every 48h in a hypo-fractionated schedule. The according oxygenation dynamics within the simulation are shown in Fig 6 for clinical accelerated schedules without fractionation-pauses during the weekend. Repeated doses of 4Gy effectively cleared the tumour of hypoxic cells for extended time periods, prior to the administration of follow-up doses. After 48h a fast rise in the hypoxic fraction led to the loss of any oxygenation-advantage for treatment, as indicated in Fig 6(b). For lower doses, 2Gy per 24h, a short-lived reoxygenation could be observed. Repeated delivery with a 24h-interval would not achieve a lasting elevation of diffusion-based hypoxia in the tumour.

Inhibition of the oxygen enhancement effect (e.g. via hypoxic sensitizers) doubled the effectiveness of irradiation to lift hypoxia in the tumour. The oxygenation response of 2Gy x-rays with *O*_max_ = 1 closely matched the response to 4Gy under the effect of normal oxygen enhancement (*O*_max_ = 2.9). Repeated irradiation with suppressed oxygen effects led to a full reoxygenation of the tumour, both for repeated delivery of 2Gy or 4Gy. For larger doses of 4Gy the delivery of only 5 fractions was sufficient in order to delay the following rise of hypoxia, so that no radiobiologically hypoxic cells were present for the following fraction.

The dynamics of hypoxia in the model spheroid illustrated, that typically the diffusion-based reoxygenation will be lost before a follow-up dose is administered in 24h and 48h intervals. When the oxygen enhancement was suppressed, a fast and persistent oxygenation of the tumour was achieved. This suggests that hypoxic sensitizers can be strategically used in treatment planing and that shorter follow-up intervals should be considered for radiotherapy.

## Discussion

Using an agent-based tumour spheroid model, we demonstrated that the temporal and spatial variations of diffusion-based, chronic hypoxia are predictable on a qualitative as well as quantitative level. The obtained results can be directly compared to *in vitro* spheroid systems or xenograft tumour models for verification or falsification, using techniques such as EPRI to track the predicted reoxygenation response [24, 81]. The same techniques can be used to obtain data which is suitable for a fine-tuning of the model to specific cell lines. The current model can provide quantitative oxygen dynamics in spheroids and micro-regions of larger tumours, which was previously hard to achieve in most models [61]. It can thus be a first step towards a prospective productive combination with quantitative imaging techniques to be used in experimental treatment planing. The choice of cell line and the according variation of parameters only affected the results on a quantitative level, while the described qualitative phenomena were conserved for all reasonable variations of the parameters. Therefore the model yields a range of generic predictions which should be observable in a wide range of tumour systems.

*In vivo*, the hypoxia dynamics will be a mixture of multiple sources of hypoxia and can be extremely diverse [25], but the isolated influence of diffusion-based hypoxia could be studied both *in vitro* and *in silico* using spheroid experiments and simulations. While the current model disregards a range of mechanisms which are undoubtedly of great importance for the treatment response of tumours, some of these mechanisms are suitable for integration within future *in silico* models. Explicit inclusion of the tumour microenvironment and the stromal background could allow for the study of resistance development. Single cell-based models can describe the selective pressure on cancer cells which is induced by hypoxic conditions and the according mutation of cells. The actions of immune agents can be directly included by a cell-based representation of immunocytes. The presented *in silico* model operates at reduced complexity but allows for a systematic, disentangled study of the influence of quiescent cells, oxygen enhancement, cell consumption and further effects on the reoxygenation dynamics. Thus a necessary simplifaction was performed as a first step on the way towards a more detailed model for experimental optimisation of treatment.

### Implications of reoxygenation dynamics on treatment

With the help of our model system we demonstrated, that the complex response of tumours to radiotherapy leads to time-windows of transient reoxygenation. It is of vital importance that this effect is investigated in further studies to be eventually considered in treatment planing. The delivery of therapeutic measures as well as imaging schedules could be adapted to this diffusion-based oxygen dynamics. Delivery should be avoided during mainly hypoxic periods and instead enforced during reoxygenated times. The reoxygenation response of tumours can only be exploited if the timing is correct. For our sample tumour system the diffusion-based reoxygenation was on a timescale which would typically conflict with common fractionation intervals of 24 hours or 48 hours. In order to employ the reoxygenation effects, the simulation results suggest the investigation of shortened delivery intervals in order to avoid re-depletion of oxygen before the next therapeutic radiation fraction is applied. This finding should be studied in *in vitro* experiments as well as in more complex *in silico* models which are to be developed.

A very fast delivery of follow-up doses has been shown to be prohibitive due to the transient rise of hypoxic survivors directly in response to irradiation. If the next dose however is delivered too late, the benefits of reoxygenation are lost. This implies, that diffusion-based hypoxia defines a cell-type dependent interval for optimal use of the reoxygenation effects. A worthwhile investigation would be the delivery of a first radiation fraction as a trigger-dose which induces reoxygenation, which is followed by a correctly timed second “effector” dose which could thus fully exploit the induced effects of reoxygenation, before they are lost due to re-hypoxation.

Reoxygenation effects however are only one of the dynamic treatment-induced changes which will influence the overall treatment sensitivity of a tumour. They will be intermixed with other treatment-induced dynamic phenomena, such as cell reactivation, cell cycle synchronisation [53], changes in tissue diffusivity and so forth. The cell cycle dependent uptake of nutrients could further affect the reoxygenation, as cell cycle resynchronisation could induce macroscopic dynamics in consumption, which would lead to the according changes in the oxygenation. As a result, it appears mandatory to connect the oxygenation effects with a model for cell cycle effects as described in [52] or [42] in order to find optimal irradiation timings in respect to more than one phenomenon. The inclusion of immune effects and of stroma are further important steps in the development of an advanced model.

The suppression of the oxygen enhancement effect for irradiation had surprisingly strong effects on the oxygenation dynamics. If the maximum oxygen enhancement *O*_max_ was set to 1, that is either a hypoxic sensitizer is in effect or the cell line inherently shows a low dependency of radiation damage on oxygenation, the higher degree of cell killing would induce a pronounced reoxygenation. Thus the oxygen-dependence of radiation damage of the specific cells is of great importance for the reoxygenation timings of the tumour.

Any radio-sensitisation of hypoxic cells prior to treatment using hypoxic sensitizers could greatly enhance the reoxygenation which results from the radiation dose within the model. Even though clinical radiosensitizer trials have been mostly inconclusive, from no effect to a clear overall benefit, it seems that they can be used to increase the reoxygenation which can be induced by irradiation.

If radiation therapies are expected to fail to achieve a reoxygenation, this has to be known in advance. Other adequate measures to re-oxygenate radioresistant hypoxic regions, such as hypoxic sensitizers or hypoxia-specific therapies to eradicate hypoxic, radioresistant cell populations [8, 82], can be successfully employed in this case. This shows a high potential to combine hypoxic sensitizers and hypoxic cytotoxins with fractionated irradiation schedules to a more holistic treatment protocol.

A combination of hypoxic cytotoxins and irradiation seems to be fruitful, as hypoxia is not fully lifted by irradiation alone and the hypoxic cell population quickly rises again after treatment. At the same time, hypoxic cytotoxins can be administered as an adjuvant pre-treatment, in order to induce cell death in hypoxic regions, which in turn will re-oxygenate the tumour and increase the radiation efficiency.

As repeated cycles of oxygenation and hypoxia have been shown to increase the aggressiveness of tumours [4, 5], it may be beneficial to optimise treatment schedules in order to avoid predictable transient cycling reoxygenation and instead aim for a constant increase in oxygen concentration. This tactic could limit the development of aggressive strategies by the tumour in response to treatment [21]. Cell-based *in silico* models are well-suited to study this question, as mutation and the resulting increased aggressiveness can be included explicitly. Predictable oxygenation changes in response to radiotherapy also open up the possibility for a subsequent targeted therapy, which would be specifically based on altered gene expression [7].

The dynamics of hypoxia clearly show, that the results of PET-scanning will critically depend on the timing of imaging, especially when imaging is combined with therapy. Imaging-techniques must not only be used as initial prognostic marker, but must instead be used as active observable in order to update and optimise treatment delivery. Active radiation planing with OER dose-painting approaches must consequently not ignore the temporal variation of hypoxia on the finer scale of hours or even minutes [83]. A combination of imaging techniques and repeated, dynamic acquisition is necessary to tackle the “complexity of underlying tumour biology” [20, 84].

As neither patients nor tumours are static systems, adaptive radiotherapy and the live update of treatment plans has become a goal in clinical treatment. However, this requires an in-detail understanding of fundamental processes which happen within the tumour in response to external stimuli, such as radio- or chemotherapy. It has been stated regularly, that the spatio-temporal oxygen dynamics during radiotherapy need to be understood in order to predict an optimal timing of treatment and continued imaging [20, 30]. Spatio-temporal mathematical models, as proposed here, can be used to further this understanding and test the reaction of tumours under full control of all parameters. In combination with imaging such models can be used for the prediction of oxygen dynamics out of range of current temporal or spatial imaging resolution [36].

## Supporting Information

S1 Fig Complex oxygen dynamics in response to irradiation with 4Gy and *O*_max_ = 1. The local oxygen concentration in the spheroid varies in dependence on cell cycle status (quiescence), tissue density and cell death. Due to the dynamic interplay of these phenomena a complex fluctuation of oxygenation levels is observed in response to treatment. Oxygen levels have been colour-coded as in Fig 3(c) and 3(d).
(TIFF)
