# Supplementary figures and images for: Spatio-Temporal Dynamics of Hypoxia during Radiotherapy
Source: PLoS One. 2015 Aug 14;10(8):e0133357. doi: 10.1371/journal.pone.0133357 (PMC4537194; doi:10.1371/journal.pone.0133357)

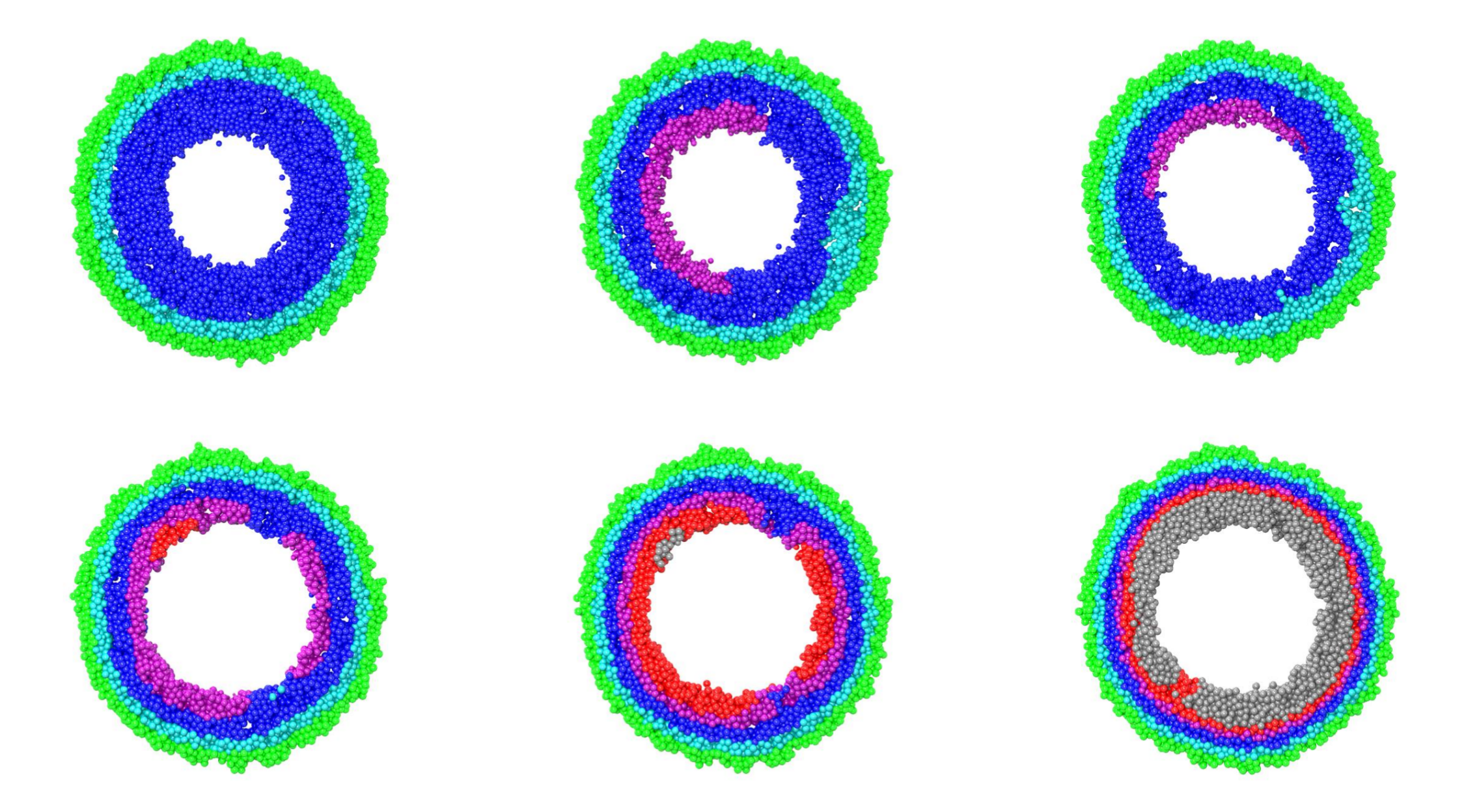

Supplement: S1 Fig — The local oxygen concentration in the spheroid varies in dependence on cell cycle status (quiescence), tissue density and cell death. Due to the dynamic interplay of these phenomena a complex fluctuation of oxygenation levels is observed in response to treatment. Oxygen levels have been colour-coded as in Fig 3(c) and 3(d). (TIFF) [file pone.0133357.s001.tiff]
